# Gestational weight gain in 4 low- and middle-income countries and associations with birth outcomes: a secondary analysis of the Women First Trial

**DOI:** 10.1093/ajcn/nqab086

**Published:** 2021-04-19

**Authors:** Melissa S Bauserman, Carla M Bann, K Michael Hambidge, Ana L Garces, Lester Figueroa, Jamie L Westcott, Jackie K Patterson, Elizabeth M McClure, Vanessa R Thorsten, Sumera Ali Aziz, Sarah Saleem, Robert L Goldenberg, Richard J Derman, Veena Herekar, Manjunath Somannavar, Marion W Koso-Thomas, Adrien L Lokangaka, Antoinette K Tshefu, Nancy F Krebs, Carl L Bose, The Women First Preconception Trial Study Group, Shivaprasad Goudar, Sangappa Dhaded, Bhalchandra Kodkany, Omrana Pasha, Abhik Das, Menachem Miodovnik, N K Raju Tonse

**Affiliations:** Department of Pediatrics, University of North Carolina at Chapel Hill, Chapel Hill, NC, USA; RTI International, Durham, NC, USA; Section of Nutrition, Department of Pediatrics, University of Colorado School of Medicine, Aurora, CO, USA; INCAP (Institute of Nutrition of Central America and Panama), Guatemala City, Guatemala; INCAP (Institute of Nutrition of Central America and Panama), Guatemala City, Guatemala; Section of Nutrition, Department of Pediatrics, University of Colorado School of Medicine, Aurora, CO, USA; Department of Pediatrics, University of North Carolina at Chapel Hill, Chapel Hill, NC, USA; RTI International, Durham, NC, USA; RTI International, Durham, NC, USA; Department of Community Health Sciences, Aga Khan University, Karachi, Pakistan; Department of Community Health Sciences, Aga Khan University, Karachi, Pakistan; Department of Obstetrics and Gynecology, Columbia University, New York, NY, USA; Department of Global Affairs, Thomas Jefferson University, Philadelphia, PA, USA; KLE Academy of Higher Education and Research's Jawaharlal Nehru Medical College, Belagavi, Karnataka, India; KLE Academy of Higher Education and Research's Jawaharlal Nehru Medical College, Belagavi, Karnataka, India; National Institute of Child Health and Human Development/NIH, Bethesda, MD, USA; Kinshasa School of Public Health, Kinshasa, Democratic Republic of the Congo; Kinshasa School of Public Health, Kinshasa, Democratic Republic of the Congo; Section of Nutrition, Department of Pediatrics, University of Colorado School of Medicine, Aurora, CO, USA; Department of Pediatrics, University of North Carolina at Chapel Hill, Chapel Hill, NC, USA; KLE Academy of Higher Education and Research's Jawaharlal Nehru Medical College, Belagavi, India; Aga Khan University, Pakistan; RTI International, North Carolina, USA; Pregnancy and Perinatology Branch, National Institute of Child Health and Human Development/NIH, USA; Pregnancy and Perinatology Branch, National Institute of Child Health and Human Development/NIH, USA

**Keywords:** gestational weight gain, developing countries, fetal development, nutrition during pregnancy, infant nutrition disorders, malnutrition, low birth weight

## Abstract

**Background:**

Adequate gestational weight gain (GWG) is essential for healthy fetal growth. However, in low- and middle-income countries, where malnutrition is prevalent, little information is available about GWG and how it might be modified by nutritional status and interventions.

**Objective:**

We describe GWG and its associations with fetal growth and birth outcomes. We also examined the extent to which prepregnancy BMI, and preconception and early weight gain modify GWG, and its effects on fetal growth.

**Methods:**

This was a secondary analysis of the Women First Trial, including 2331 women within the Democratic Republic of Congo (DRC), Guatemala, India, and Pakistan, evaluating weight gain from enrollment to ∼12 weeks of gestation and GWG velocity (kg/wk) between ∼12 and 32 weeks of gestation. Adequacy of GWG velocity was compared with 2009 Institute of Medicine recommendations, according to maternal BMI. Early weight gain (EWG), GWG velocity, and adequacy of GWG were related to birth outcomes using linear and Poisson models.

**Results:**

GWG velocity (mean ± SD) varied by site: 0.22 ± 0.15 kg/wk in DRC, 0.30 ± 0.23 in Pakistan, 0.31 ± 0.14 in Guatemala, and 0.39 ± 0.13 in India, (*P *<0.0001). An increase of 0.1 kg/wk in maternal GWG was associated with a 0.13 cm (95% CI: 0.07, 0.18, *P *<0.001) increase in birth length and a 0.032 kg (0.022, 0.042, *P *<0.001) increase in birth weight. Compared to women with inadequate GWG, women who had adequate GWG delivered newborns with a higher mean length and weight: 47.98 ± 2.04 cm compared with 47.40 ± 2.17 cm (*P *<0.001) and 2.864 ± 0.425 kg compared with 2.764 ± 0.418 kg (*P *<0.001). Baseline BMI, EWG, and GWG were all associated with birth length and weight.

**Conclusions:**

These results underscore the importance of adequate maternal nutrition both before and during pregnancy as a potentially modifiable factor to improve fetal growth.

## Introduction

Adequate maternal nutrition is an important component of the “first 1000 days,” a critical time to promote healthy child growth. Gestational weight gain (GWG) is one measure of maternal nutrition status, and appropriate GWG is essential for healthy fetal growth and birth outcomes (1–3). Inappropriate (inadequate or excessive) GWG might have a lasting impact beyond the neonatal period, by influencing growth in the offspring throughout early childhood (4, 5). Despite the association between GWG and healthy fetal growth, information is limited with regards to GWG and postnatal growth in low- and middle-income countries (LMICs) where childhood malnutrition is a major cause of mortality. In these contexts, women might not be weighed as part of routine antenatal care, counseled about healthy GWG, nor have access to adequate food to modify nutritional intake (6).

The Women First Trial evaluated the impact of a maternal nutrition supplement initiated either before or early in pregnancy on length at birth in 4 LMICs (7). This study showed an improvement in birth length and weight when mothers were given the nutritional supplement compared with a control arm. The nutritional supplement might have influenced GWG among participants and might have a differential effect based on maternal nutritional status, as measured by maternal weight and BMI at enrollment, prior to conception (8–11). GWG has not been widely studied in low-resource settings and might be an important mediator of the effects nutritional supplements have on fetal growth.

In these secondary analyses of participants in the Women First Trial, we describe GWG and its associations with fetal growth and birth outcomes in 4 LMICs. We describe GWG within each country by baseline maternal nutritional status and the receipt of nutrition interventions. We explore the interactions between baseline BMI status and GWG. We also evaluate how GWG might mediate the effects of maternal nutrition interventions.

## Methods

The details of the Women First Trial have been previously reported (12). Briefly, this was an individually randomized, nonmasked, multisite, controlled efficacy trial conducted in research sites in the Democratic Republic of Congo (DRC), Guatemala, India, and Pakistan. The primary aim of this trial was to identify the effects of maternal nutrition supplementation at 2 different time points: preconception (Arm 1), at the end of the first trimester (Arm 2), and no supplementation (Arm 3, control) on birth length. Women were included if they were aged 16–35 y, parity 0–5, and expecting to become pregnant. We excluded nulliparous women who were unwilling to deliver in a hospital and those who were severely anemic. Women were randomly assigned by permuted block design with stratification by research cluster, allocating women in a 1:1:1 ratio within the 3 treatment arms (12).

Women who were randomly assigned to Arm 1 started a small quantity, lipid-based micronutrient supplement (supplement 1) from the time of randomization until delivery. Women who received ≥3 mo of supplement prior to conception were included. Women randomly assigned to Arm 2 received the same lipid-based micronutrient supplement, beginning at the end of the first trimester through to delivery. Women in Arms 1 or 2 that had a BMI <20 kg/m^2^ or suboptimal GWG, based on the Institute of Medicine (IOM) recommendations, received an additional protein-energy nutrition supplement (supplement 2) when they started nutrition supplementation. Women randomly assigned to Arm 3 did not receive nutrition supplementation from the study at any point.

Trained assessment teams measured women and newborns using standardized equipment and procedures. Maternal height and weight were measured at enrollment. We obtained maternal weight measurements at 2 additional times during gestation, ∼12 weeks and 32 weeks of gestation. This report includes all women from the Women First Trial who had 2 measurements for weight and for whom birth outcomes were available. We calculated baseline BMI based on height and weight measured at enrollment, prior to conception. The time interval between enrollment and conception varied, therefore, we evaluated the change in weight rather than the weight gain velocity in the preconception and early gestation interval. We defined baseline measurements for BMI as underweight (BMI <18.5 kg/m^2^), normal weight (18.5–24.9), overweight (25.0–29.9), and obese (≥30) (13). As a small percentage (4% of the overall sample) of mothers were obese, we combined obese mothers with overweight mothers in the analyses.

Newborn anthropometry was measured within 48 h after delivery. Newborn weight was measured in triplicate using a seca 334 electronic scale, and newborn length was measured using a neonatal stadiometer (Ellard Instrumentation Ltd). We defined low birth weight (LBW) as a newborn weight <2500 g. Gestational age was determined by ultrasound when available, using crown-rump length measurements within the first trimester. Ultrasound dating was not available for all participants, therefore, we excluded data from women without ultrasound dating from outcome analyses that depended upon precise assignment of gestational age. No ultrasounds were performed in the DRC.

We defined GWG velocity as the change in weight (kg) between the 2 gestational weight measurements, divided by the number of weeks between the 2 measurements. We chose this method because it was not dependent on the precise assessment of gestational age at the time of the measurements. Secondarily, we repeated the analyses on a subset of women for whom gestational dating by ultrasound was performed, using alternate definitions for GWG. For this subset, we assigned each woman a Z-score based on her GWG and number of weeks gestation using INTERGROWTH-21st standards (14). For these analyses, we defined anthropometry at birth by length-for-age Z-score (LAZ) and weight-for-age Z-score (WAZ), based on INTERGROWTH-21^st^ standards (15). We examined distributions of the variables and conducted diagnostic tests to assess model assumptions (e.g., normal distribution).

We evaluated the “adequacy” of GWG velocity based on the 2009 IOM recommendations for GWG velocity in the second and third trimesters (4). We chose the IOM recommendations since there are no consensus recommendations for adequate weight gain during pregnancy for populations in LMICs. We categorized a woman as having inadequate GWG velocity if her GWG velocity was below the lower limits of the IOM recommendations, or <0.51 kg/wk for an underweight woman (baseline BMI <18.5); <0.42 kg/wk for a normal weight woman (baseline BMI 18.5–24.9); <0.28 kg/wk for an overweight woman (baseline BMI 25.0–29.9); and <0.22 kg/wk for an obese woman (baseline BMI ≥30). We categorized a woman as having “excess” GWG velocity if her GWG velocity was above the upper limits of the IOM recommendations, or >0.58 kg/wk for an underweight woman; >0.50 kg/wk for a normal weight woman; >0.33 kg/wk for an overweight woman; and >0.27 kg/wk for an obese woman.

For the statistical analyses, we began by conducting ANOVA to compare mean GWG velocity by treatment arm and demographic characteristics (site, baseline BMI, age, and parity). We conducted chi-square tests of treatment arm and demographic characteristics by adequacy of GWG. We then fitted mixed effect regression models of the outcomes by GWG velocity, using linear models to estimate adjusted mean differences for continuous outcomes (newborn weight and length) and Poisson models to estimate adjusted relative risks for categorical outcomes (LBW and preterm birth). Each model controlled for treatment arm, demographic characteristics, and weight gain from baseline to 12 weeks of gestation, and accounted for clustering of participants by sampling clusters within sites. We performed sensitivity analyses by including and excluding adolescent mothers in the models.

We evaluated the effect of weight gain from baseline (prior to conception) to 12 wk (termed “preconception and early weight gain, or EWG”) and conducted bivariable analyses to determine if EWG was associated with GWG or birth outcomes [length, weight, preterm birth, LBW, or small-for-gestational age (SGA)]. A conditional linear regression model was used to examine the independent association for the different weight gain intervals and birth outcomes (16).

Next, the associations between adequate GWG and birth outcomes were examined, and whether these associations varied by baseline BMI category (underweight, normal weight, and overweight/obese). We compared outcomes for women with adequate, inadequate, or excessive weight gain, using t-tests for continuous outcomes and chi-square tests for categorical outcomes. Then, we fitted mixed effect regression models to compare outcomes by adequacy of weight gain controlling for treatment arm and demographics and accounting for sampling clusters. These analyses were conducted for all participants and separately by BMI category. The descriptive analyses and regression models were conducted using SAS version 9.4 (SAS Institute).

Finally, we conducted mediation analyses to test whether GWG served as a mediator between treatment arm and outcomes. As treatment arm is a multicategory nominal variable, we used the approach described by Hayes and Preacher for mediation analyses with multicategorical independent variables (17). The mediation analyses were conducted in the structural equation modeling framework with Mplus version 8.3 (Muthén & Muthén), using bootstrapping to determine CIs.

The Women First Trial was approved by the Colorado Multiple Institutional Review Board, University of Colorado, the local and/or national ethics committees at each research site, and the data coordinating center. All participants provided verbal and written consent to be included in the trial. The Women First Trial was registered at clinicaltrials.gov as NCT01883193 and the study protocol is published online (18).

## Results

**FIGURE 1:**
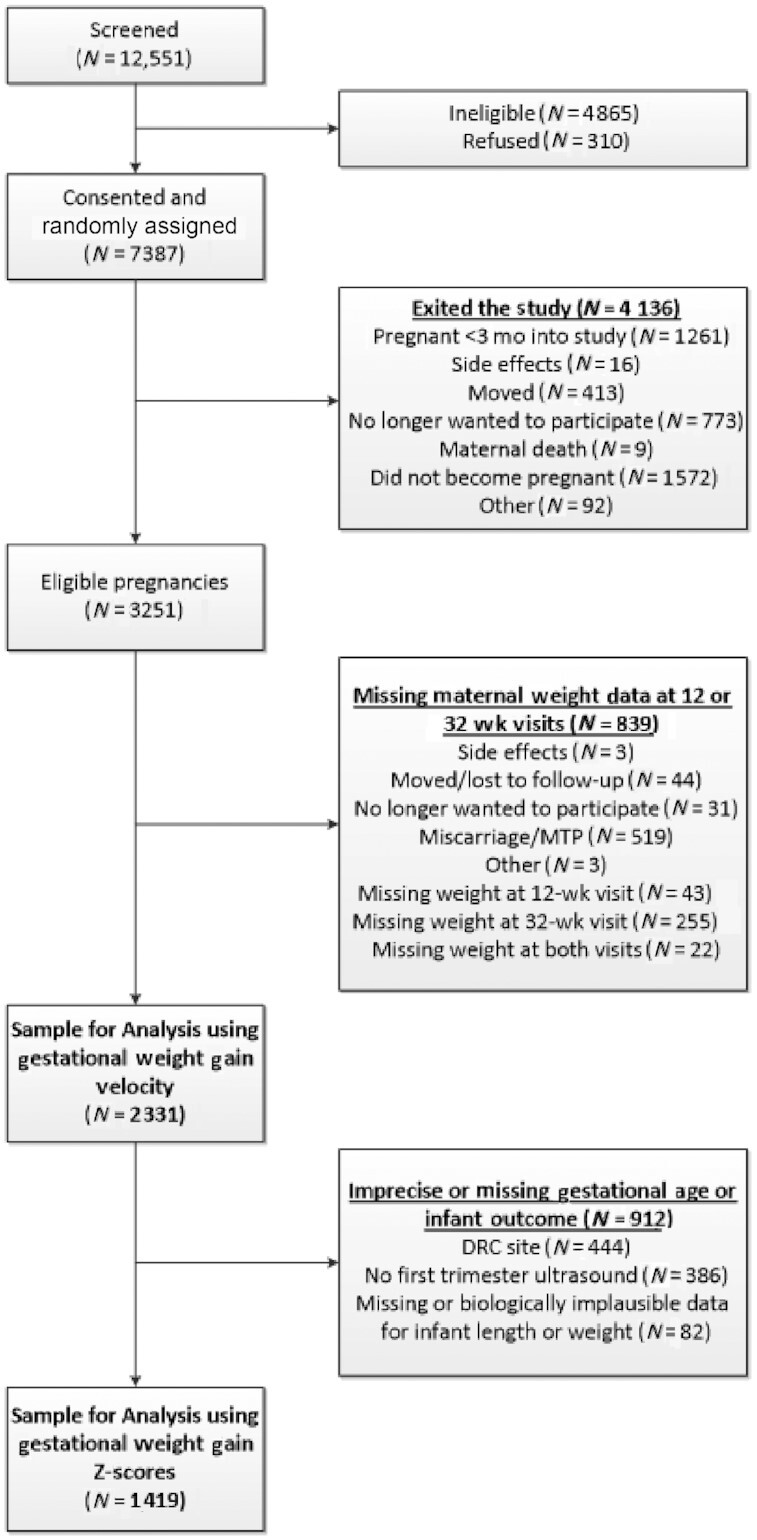
Consolidated Standards of Reporting Trials (CONSORT) diagram of participant flow. Overall screening, randomization, and obtainment of primary outcome. DRC, Democratic Republic of Congo; MTP, medical termination of pregnancy.

We report 2331 women from the Women First Trial (95% of women with the primary outcome) from research sites in DRC, Guatemala, India, and Pakistan (**Figure 1**). Roughly equal numbers of women within each site were randomly assigned to each study arm (**Table 1**). Mean (± SD) baseline BMI for the sample was 21.5 ± 4.1, however, the mean varied from 19.7 ± 2.9 in the Pakistani site to 25.5 ± 4.2 in the Guatemalan site (Table 1). Overall, 23% of the sample was underweight based on baseline BMI. However, this percentage also varied by site, with only 1% underweight in the Guatemalan site and 35% and 37% underweight in the Pakistani and Indian sites, respectively. Only 4% of the overall sample was obese, based on baseline BMI, but this percentage also varied by site, with 1% obese in the DRC, Indian, and Pakistani sites, and 14% obese in the Guatemalan site. Maternal age distribution was similar in all sites and 21% of the sample were nulliparous (Table 1).

**TABLE 1:** Baseline maternal demographics combined and by site

Characteristic	All sites (*n *= 2331)	DRC (*n *= 444)	Guatemala (*n *= 611)	India (*n *= 590)	Pakistan (*n *= 686)
Treatment Arm^1^					
Arm 1	756 (32)	143 (32)	179 (29)	192 (33)	242 (35)
Arm 2	825 (35)	162 (36)	225 (37)	201 (34)	237 (35)
Arm 3	750 (32)	139 (31)	207 (34)	197 (33)	207 (30)
Maternal weight, kg	48.8 ± 9.0	50.2 ± 7.8	54.0 ± 9.8	45.9 ± 8.4	45.7 ± 7.1
Baseline BMI, kg/m^2^ (mean ± SD)	21.5 ± 4.1	20.6 ± 2.6	25.5 ± 4.2	20.0 ± 3.4	19.7 ± 2.9
Baseline BMI categories					
Underweight (<18.5)	542 (23)	81 (18)	6 (1)	217 (37)	238 (35)
Normal (18.5–24.9)	1394 (60)	344 (77)	310 (51)	325 (55)	415 (61)
Overweight (25.0–29.9)	293 (13)	15 (3)	210 (34)	40 (7)	28 (4)
Obese (≥30)	101 (4)	4 (1)	84 (14)	8 (1)	5 (1)
Age, y (mean ± SD)	23.3 ± 4.2	22.8 ± 4.5	24.3 ± 4.4	21.9 ± 3.4	23.7 ± 4.1
Age, y					
<20	475 (20)	114 (26)	91 (15)	146 (25)	124 (18)
20–24	962 (41)	167 (38)	242 (40)	323 (55)	230 (34)
25+	894 (38)	163 (37)	278 (46)	121 (21)	332 (48)
Parity					
Nulliparous	486 (21)	92 (21)	42 (7)	148 (25)	204 (30)
Primi/multiparous	1845 (79)	352 (79)	569 (93)	442 (75)	482 (70)

Data presented as *n* (%) unless otherwise noted.

1 Maternal participants in Arm 1 started the intervention ≥3 mo prior to conception; Arm 2 started the same intervention at ∼12 weeks of gestation; and Arm 3 (control) received no study intervention.

DRC, Democratic Republic of Congo.

GWG velocity varied by site, *P *<0.001 (**Table 2**). The DRC site had the lowest mean (± SD) GWG velocity at 0.22 ± 0.15 kg/wk and the Indian site had the highest GWG velocity at 0.39 ± 0.13 kg/wk. There was no significant difference noted in GWG velocity by treatment arm (*P *= 0.10). We noted differences in GWG velocity by baseline BMI category, age, and parity. Women with lower baseline BMIs had higher GWG velocities: 0.33 ± 0.17 kg/wk for underweight women; 0.31 ± 0.18 kg/wk for normal weight women; and 0.28 ± 0.18 kg/wk for overweight/obese (*P *<0.001). GWG velocity also differed by maternal age: 0.29 ± 0.19 kg/wk for women aged<20 y; 0.33 ± 0.17 kg/wk for women aged 20–24 y, and 0.30 ± 0.18 kg/wk for women aged 25 y or older (*P *<0.001). Nulliparous women had lower GWG velocity (0.29 ± 0.20 kg/wk) than multiparous women (0.31 ± 0.18 kg/wk) (*P *<0.05). Results were similar when repeated without adolescent mothers included in the model.

**TABLE 2:** Gestational weight gain velocity and adequacy by maternal demographics

	Gestational weight gain velocity^1^ (kg/wk)			Adequacy of gestational weight gain (*n* [%])			
Variable	*n*	Mean ± SD	*P* value	Inadequate (*n *= 1731)	Adequate (*n* = 242)	Excessive (*n *= 345)	*P* value
Site							
DRC	440	0.216 ± 0.154	<0.001	410 (93)	13 (3)	17 (4)	<0.001
Guatemala	610	0.307 ± 0.144		386 (63)	77 (13)	146 (24)	
India	589	0.392 ± 0.125		396 (67)	79 (13)	114 (19)	
Pakistan	680	0.298 ± 0.228		539 (79)	73 (11)	68 (10)	
Treatment Arm^2^							
Arm 1	753	0.307 ± 0.178	0.10	584 (78)	65 (9)	104 (14)	0.086
Arm 2	822	0.319 ± 0.180		588 (72)	99 (12)	134 (16)	
Arm 3	744	0.299 ± 0.182		559 (75)	78 (10)	107 (14)	
Baseline BMI categories^3^							
Underweight	541	0.328 ± 0.172	<0.001	483 (89)	22 (4)	36 (7)	<0.001
Normal weight	1384	0.310 ± 0.184		1058 (76)	176 (13)	150 (11)	
Overweight/obese	393	0.277 ± 0.175		190 (48)	44 (11)	159 (40)	
Age, y							
<20	474	0.292 ± 0.195	<0.001	366 (77)	44 (9)	64 (14)	0.62
20–24	955	0.326 ± 0.172		700 (73)	107 (11)	147 (15)	
25+	890	0.300 ± 0.180		665 (75)	91 (10)	134 (15)	
Parity							
Nulliparous	482	0.294 ± 0.196	0.038	376 (78)	40 (8)	66 (14)	0.13
Primi/multiparous	1837	0.313 ± 0.176		1355 (74)	202 (11)	279 (15)	

Data presented as mean ± SD or *n* (%). ANOVA were used for weight gain comparisons and chi-square tests for adequacy of weight gain comparisons.

1 Gestational weight gain velocity was defined as the change in weight (kg) between 2 maternal weight measurements, divided by the number of weeks between the 2 measurements. Maternal weight measurements were recorded at ∼12 and 32 weeks of gestation.

2 Maternal participants in Arm 1 started the intervention ≥3 mo prior to conception; Arm 2 started the same intervention at ∼12 weeks of gestation; and Arm 3 (control) received no study intervention.

3 Baseline BMI categories were defined based on the maternal weight at enrollment in the study, prior to conception: Underweight (<18.5), Normal Weight (18.5–24.9), and Overweight/Obese (≥25).

DRC, Democratic Republic of Congo.

The interval of EWG varied from 8 to 116 wk (1 participant had a pregnancy that occurred before 12 wk, which was a protocol violation) based on the timing of conception after baseline measurements were obtained. After adjusting for baseline demographic features (maternal age, parity, and baseline BMI), women gained a mean (95% CI) of 0.86 kg (0.54, 1.19 kg) in Arm 1, 0.08 (−0.24, 0.40) in Arm 2, and 0.31 (−0.02, 0.65) in Arm 3, (control). Women in Arm 1 had more EWG than women in Arm 2 (*P* <0.001) and Arm 3 (*P *= 0.003).

EWG was associated with GWG velocity [regression coefficient 0.01 (95% CI: −0.02, −0.01), *P *<0.001]. The independent associations of interval weight gain and birth length and weight are shown in **Table 3**. Higher maternal weight at baseline and larger maternal weight gain in the EWG period were associated with longer birth lengths and larger birth weights. This association pattern was demonstrated among underweight, normal weight, overweight, and obese mothers. EWG was associated with birth length [RR 0.04 (95% CI: 0.02, 0.07), *P* <0.001], weight [RR 0.01 (95% CI: 0.01, 0.02), *P *<0.001], LBW [RR 0.97 (95% CI: 0.95, 0.99), *P *= 0.003], and SGA [RR 0.97 (95% CI: 0.95, 0.99), *P *= 0.006], after controlling for baseline demographics.

**TABLE 3:** Newborn length and weight by baseline maternal weight status, early weight gain (EWG, baseline to 12 wk), and gestational weight gain (GWG, 12–32 wk)

BMI category^1^	Length, cm		Weight, kg	
Maternal weight^2^	B (95% CI)	*P*	B (95% CI)	*P*
All participants				
Baseline weight	0.06 (0.05, 0.07)	<0.001	0.014 (0.012, 0.016)	<0.001
EWG	0.09 (0.06, 0.12)	<0.001	0.021 (0.016, 0.025)	<0.001
GWG	0.09 (0.07, 0.12)	<0.001	0.021 (0.016, 0.026)	<0.001
Underweight				
Baseline weight	0.12 (0.07, 0.17)	<0.001	0.021 (0.013, 0.030)	<0.001
EWG	0.13 (0.07, 0.19)	<0.001	0.027 (0.016, 0.038)	<0.001
GWG	0.15 (0.09, 0.21)	<0.001	0.030 (0.020, 0.041)	<0.001
Normal weight				
Baseline weight	0.09 (0.07, 0.11)	<0.001	0.019 (0.015, 0.023)	<0.001
EWG	0.08 (0.05, 0.11)	<0.001	0.020 (0.014, 0.026)	<0.001
GWG	0.08 (0.05, 0.12)	<0.001	0.020 (0.013, 0.026)	<0.001
Overweight/Obese				
Baseline weight	0.04 (0.01, 0.06)	0.002	0.006 (0.000, 0.011)	0.019
EWG	0.08 (0.03, 0.13)	0.002	0.016 (0.005, 0.026)	0.004
GWG	0.06 (0.01, 0.12)	0.020	0.013 (0.001, 0.024)	0.032

Note: Regression coefficients (B) are based on a linear mixed effects regression model accounting for site, study arm, maternal age, and parity.

1 Baseline BMI categories were defined based on the maternal weight at enrollment into the study, prior to conception. Categories were defined as: Underweight (<18.5 kg/m^2^), Normal Weight (18.5–24.9), and Overweight/Obese (≥25).

2 Baseline weight is the maternal weight at enrollment into the study. Early weight gain (EWG) is the change in maternal weight (kg) between the interval of baseline and 12 wk. Gestational weight gain (GWG) is the change in maternal weight (kg) between 12 and 32 weeks of gestation.

We identified 74% of women as having inadequate GWG, 10% with adequate weight gain, and 15% of women with excessive weight gain. Inadequate weight gain was common in all sites, ranging from 63% of women in Guatemala to 93% of women in the DRC (Table 2). Excessive weight gain was uncommon in the DRC site (4% of women), but more common in the Indian (19%) and Guatemalan (24%) sites. There was no significant difference in adequacy of GWG by treatment arm (*P *= 0.086) and no differences in adequacy of GWG based on maternal age or parity.

**FIGURE 2:**
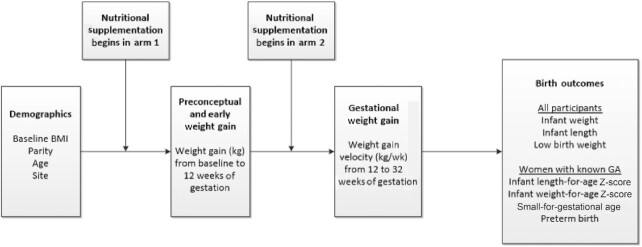
Theoretical framework. Framework used to incorporate interventions and variables into statistical models to determine relations between exposures and outcomes. This framework is limited to the variables included in this analysis and does not include other potentially important confounders. GA, gestational age.

The theoretical framework describing GWG as the exposure in a series of models controlling for pertinent confounders is displayed in **Figure 2**. After controlling for treatment arm, age, parity, and EWG, an increase in GWG was positively associated with increases in newborn length and weight across all sites. Based on our regression model, an increase of 0.10 kg/wk of maternal GWG was associated with a 0.13 cm (95% CI: 0.07, 0.18, *P *<0.001) increase in birth length and a 0.032 kg (95% CI: 0.02, 0.04, *P *<0.001) increase in birth weight. We report an 8% reduction in LBW with an increase of 0.1 kg/wk GWG velocity [RR 0.92 (95% CI: 0.88, 0.96), *P *<0.001].

When women without ultrasound-based gestational age assignment were excluded from analyses (Figure 1) and INTERGROWTH-21^st^ standards were used to evaluate GWG (using INTERGROWTH-21^st^ Z-scores for maternal weight gain) and newborn anthropometry (using LAZ and WAZ based on INTERGROWTH-21^st^ Z-scores), we observed similar associations (**Supplemental Table 1**). Using INTERGROWTH-21^st^ standards, we noted differences in GWG velocity by site, treatment arm, baseline BMI category, and age but not parity (**Supplemental Table 2**). An increase of 1 SD in maternal weight gain Z-score was associated with an increase of 0.06 SD in newborn LAZ (*P* <0.01) and an increase of 0.05 SD in newborn WAZ (*P* <0.01). Whereas no significant difference in SGA [adj. RR 0.96 (95% CI: 0.92, 1.00)] was observed, we found a reduced risk of preterm birth [adj. RR 0.87 (95% CI: 0.79, 0.86)] with increasing gestational weight Z-scores (Supplemental Tables 1 and 2).

**FIGURE 3:**
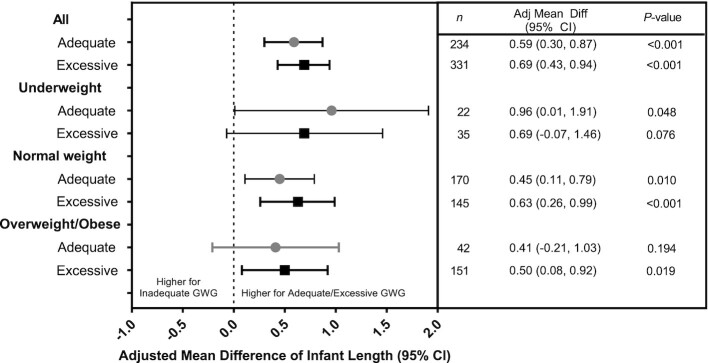
Adjusted mean differences of infant length by adequacy of gestational weight gain (GWG). Values are mean differences in infant weight (kg) between those with adequate or excessive compared with inadequate GWG after accounting for site, study arm, maternal age, parity, and weight gain from baseline to 12 weeks of gestation using a generalized linear mixed effects model. Weight categories are defined based on BMI: underweight (BMI <18.5), normal weight (BMI 18.5–24.9), and overweight/obese (BMI ≥25). Numbers of women with inadequate GWG by baseline BMI are: underweight (*n *= 471), normal weight (*n *= 1020), and overweight (*n *= 181).

**FIGURE 4:**
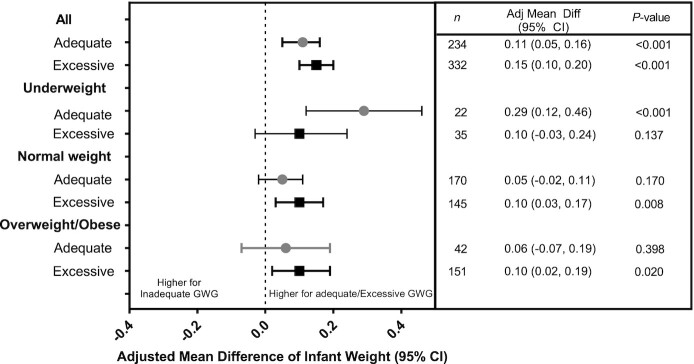
Adjusted mean differences of infant weight by adequacy of gestational weight gain (GWG). Values are mean differences in infant length (cm) between those with adequate or excessive compared with inadequate GWG after accounting for site, study arm, maternal age, parity, and weight gain from baseline to 12 weeks of gestation using a linear mixed effects model. Weight categories are defined based on BMI: underweight (BMI <18.5), normal weight (BMI 18.5–24.9), and overweight/obese (BMI ≥25). Numbers of women with inadequate GWG by baseline BMI are: underweight (*n *= 471), normal weight (*n *= 1020), and overweight (*n *= 181).

Newborn length differed among women with inadequate, adequate, and excessive GWG velocity (**Figure 3**). Women who had adequate and excessive GWG delivered newborns with higher mean newborn lengths (47.98 cm and 48.06 cm, respectively) than women with inadequate GWG (47.40 cm, *P *<0.001). The positive association between adequate and excessive GWG and newborn length was most notable among normal weight women. Women who had adequate and excessive GWG delivered newborns with higher mean newborn weights (2.864 kg and 2.901 kg, respectively) than women with inadequate GWG (2.764 kg, *P *<0.001) (**Figure 4**). The positive association between adequate GWG and newborn weight was observed for underweight women, but not normal weight or overweight/obese women.

We did not find a significant mediation effect between treatment arm and length or weight outcomes by GWG velocity between Arm 1 and the controls, Arm 3 (data not shown). We observed statistically significant mediation effects between Arms 2 and 3 with regards to length [coefficient 0.021 (95% CI: 0.003, 0.047)] and weight [coefficient 0.006 (95% CI: 0.001, 0.012)] by GWG velocity. However, we repeated these analyses using the subset of participants for which gestational age was known and using INTERGROWTH-21^st^ standards for LAZ and WAZ, and observed no significant mediating effect on LAZ [coefficient 0.009 (95% CI: −0.001, 0.027)] or WAZ [coefficient 0.011 (95% CI: 0.000, 0.027)].

## Discussion

We report significant associations between baseline BMI, EWG, and GWG velocity with the outcomes of newborn length and weight. These associations varied by baseline maternal BMI status. For all maternal weight categories, adequate GWG, according to the IOM recommendations, was associated with an 8% reduction in LBW, significant differences in newborn birth length, and for underweight women, birth weight. Although these effects are small, the positive association between GWG velocity and fetal growth underscores the importance of optimal maternal nutrition to support weight gain during pregnancy to optimize birth size, especially noting the apparent benefit of preconception weight status and EWG.

In our low-resource settings where many women are undernourished, their preconception nutritional status might affect the development and growth of the placenta and fetus through alterations in placental blood flow, alterations in the epigenetic state, or fetal programming (8, 10, 19). Low maternal BMI may also limit the effectiveness of nutrition supplementation (8). These outcomes of fetal growth might be influenced and improved with adequate GWG during pregnancy. In our study, underweight women or women with low GWG were offered additional protein-energy supplementation (supplement 2), which started prior to conception for women in Arm 1, and not before ∼11 weeks of gestation for Arm 2. However, only 11% of underweight women had adequate GWG despite the availability of this additional supplementation, emphasizing the challenge of alleviating chronic malnutrition during pregnancy using a relatively acute dietary intervention, especially in settings with food insecurity. In addition, the majority of the women in this study consumed diets with marginal intakes of energy and protein for pregnancy, and the additional study supplements may not have achieved optimal intakes (20).

We observed similar GWG velocity by treatment arm. This finding differs from the findings of the parent trial that showed modestly higher weight gain among women in Arm 1 than in the other 2 arms (7). We assume the differences in our report are related to our definition of GWG velocity for these analyses. In the parent trial, GWG was reported as absolute weight gain (kg) during pregnancy. In this study, we describe GWG as a function of time between measurements, thereby describing GWG velocity (kg/wk). This finding could have also been influenced by the manner in which we provided nutritional supplementation during the course of the Women First Trial, in which participating women were closely monitored with monthly weight checks to assess gestational weight gain. If women receiving the primary micronutrient fortified supplement (supplement 1) experienced suboptimal weight gain during the study, they were given additional supplementation (supplement 2) to improve GWG, which could have led to similar GWG among women in Arms 1 and 2. We also recognize that GWG is on the causal pathway between the nutrition supplementation and fetal growth and could mediate the relation; however, our mediation analysis did not show a significant effect.

We observed an association among preconception and EWG, treatment arm, GWG velocity, and birth outcomes. Women in Arm 1 had greater weight gain in the EWG period, coinciding with the timing of initiation of nutrition supplementation immediately after enrollment and randomization. During the preconception interval, women in Arm 1 were provided the nutritional intervention (micronutrients and modest calorie/protein supplementation) and an additional protein-energy supplement (supplement 2) if their weight was suboptimal (7). The mean weight gain in the EWG period was small (mean 0.86 kg) among women in Arm 1, controlling for other factors, but higher than for women in Arms 2 and 3 who did not receive nutrition supplementation.

Our study benefitted from the multicountry design within LMICs and from the careful measurement of newborn anthropometry and the inclusion of newborn length as a primary outcome; however, we recognize some limitations. We are limited by the timing of our assessment of GWG. Our last measurement was at ∼32 weeks of gestation, and therefore did not include potential influences of GWG during the final weeks of gestation. We used GWG velocity as the measure of GWG. Although GWG velocity is not consistent across the entire pregnancy, we evaluated 2 time points that encompassed the second trimester and the beginning of the third trimester, a period of rapidly increasing GWG. Although this measure of GWG velocity could have bias when evaluating preterm birth, we conclude that our endpoint at 32 wk reduces but does not eliminate that bias (20).

We were also limited by our inability to determine gestational age among many participants. Although we used ultrasound to confirm gestational dating in 3 sites when available, ultrasound dating was unavailable in the DRC. Therefore, we are limited in our ability to evaluate gestational-age-dependent growth variables among those women. However, when we limited our sample to the participants with known gestational age and repeated the analyses (Supplemental Tables 1 and 2), associations between markers of GWG and newborn length and weight were similar. For these supplemental analyses, we compared the observed GWG among our participants to the INTERGROWTH-21^st^ GWG standard. Although the INTERGROWTH-21^st^ standard was created using normal weight women with initial weights measured between 9 and 14 weeks of pregnancy, we applied it to our cohort that included underweight, overweight, and obese women. Similarly, references to evaluate adequacy of weight gain in more austere settings in LMICs are not available. We used the 2009 IOM recommendations for adequacy of GWG, but we recognize that these recommendations might not be applicable in low-resource settings.

In conclusion, maternal preconception BMI, maternal weight gain between randomization ≥3 mo prior to conception and 12 weeks of gestation, and GWG velocity between ∼12 and 32 weeks of gestation were all independently associated with birth length and weight. These findings underscore the importance of adequate maternal weight status prior to conception and weight gain during pregnancy. They also confirm the value of maternal nutrition supplements initiated ≥3 mo prior to conception in resource-poor populations in LMICs.

## Supplementary Material

nqab086_Supplemental_File

## Data Availability

Data described in the manuscript, codebook, and analytic code are publicly and freely available without restriction at NICHD's Data and Specimen Hub (DASH): https://dash.nichd.nih.gov/study/228833.

## Notes

Supported by the Bill & Melinda Gates Foundation (BMGF) Grant Number OPP1055867 and The *Eunice Kennedy Shriver* NICHD and the Office of Dietary Supplements, NIH U10 HD 076474. BMGF did not have a role in the design, implementation, analyses, or interpretation of the data.

Supplemental Tables 1 and 2 are available from the “Supplementary data” link in the online posting of the article and from the same link in the online table of contents at https://academic.oup.com/ajcn/.

NFK and CLB share senior authorship.

Abbreviations used: DRC, Democratic Republic of Congo; EWG, early weight gain; GWG, gestational weight gain; IOM, Institute of Medicine; LAZ, length-for-age Z-score; LBW, low birth weight; LMIC, low- and middle-income country; SGA, small-for-gestational age; WAZ, weight-for-age Z-score.
