# Supplementary material for: Gestational weight gain in 4 low- and middle-income countries and associations with birth outcomes: a secondary analysis of the Women First Trial
Source: Am J Clin Nutr. 2021 Apr 19;114(2):804–12. doi: 10.1093/ajcn/nqab086 (PMC8326045; doi:10.1093/ajcn/nqab086)
Supplement: nqab086_Supplemental_File [file nqab086_supplemental_file.pdf]

Title: Gestational weight gain in four low- and middle-income countries in the Women First Trial and associations with birth outcomes
First Author: Melissa Bauserman
Online Supplemental Material

Supplemental Table 1. Gestational weight gain (GWG) velocity and Z-scores by demographics in subset of sample with accurate gestational age dating

**Variable**	N	Weight Gain Velocity[1] (kg/wk)	Weight Gain Z-score[2]
Site			
Guatemala	479	0.30 ± 0.14***	-1.31 ± 1.14***
India	493	0.38 ± 0.12	-0.62 ± 0.81
Pakistan	447	0.30 ± 0.18	-1.45 ± 1.80
DRC[3]	NA	NA	NA
Treatment Arm[4]			
Arm 1	471	0.33 ± 0.15*	-1.10 ± 1.29*
Arm 2	505	0.34 ± 0.15	-1.02 ± 1.34
Arm 3	443	0.31 ± 0.16	-1.23 ± 1.42
Baseline BMI Categories			
Underweight (<18.5 kg/m^2)	358	0.35 ± 0.14***	-0.96 ± 1.38***
Normal (18.5-24.9 kg/m^2)	784	0.34 ± 0.15	-1.02 ± 1.30
Overweight/Obese(≥25 kg/m^2)	276	0.27 ± 0.16	-1.58 ± 1.37
Age, y			
<20	292	0.33 ± 0.16**	-1.08 ± 1.68**
20-24	599	0.34 ± 0.15	-0.99 ± 1.23
25+	528	0.31 ± 0.15	-1.27 ± 1.26
Parity			
Nulliparous	295	0.32 ± 0.17	-1.17 ± 1.62
Primi/Multiparous	1124	0.33 ± 0.15	-1.10 ± 1.27

Data presented as mean ± SD. Analysis of variance was used for comparisons.
**P* <0.05, **P*<0.01, ****P*<0.001 for overall F-test.

[1]Weight gain velocity was defined as the change in weight (kg) between 2 maternal weight measurements, divided by the number of weeks between the two measurements.
[2]Weight gain Z-score was assigned based on GWG and number of weeks gestation using INTERGROWTH-21st International Standards for Newborn Size (1).
[3]Democratic Republic of the Congo excluded due to imprecise gestational dating.
[4]Maternal participants in Arm 1 started intervention ≥3months prior to conception; Arm 2 started same intervention at ~12 wk gestation; and Arm 3 (control) received no study intervention.
kg/wk, kilograms/wk.

Supplemental Table 2. Newborn outcomes based on INTERGROWTH-21st International Standards (1) by gestational weight Z-score in in subset of sample with accurate gestational age dating by maternal weight category

Outcome	All Sites[1] (N=1,419) Adj. Mean Dif/RR[2,3] (95% CI)	Guatemala (N=479) Adj. Mean Diff/RR (95% CI)	India (N=493) Adj. Mean Diff./RR (95% CI)	Pakistan (N=447) Adj. Mean Diff/RR (95% CI)
**Continuous Outcomes**				
Length-for-Age Z-score				
All weight categories[4]	0.06 (0.01, 0.10)**	0.14 (0.07, 0.21)***	0.24 (0.12, 0.35)***	-0.03 (-0.09, 0.04)
Underweight	0.10 (0.01, 0.18)*	Non-estimable	0.30 (0.10, 0.50)**	0.07 (-0.03, 0.18)
Normal weight	0.06 (0.00, 0.12)*	0.17 (0.05, 0.29)**	0.31 (0.16, 0.45)***	-0.06 (-0.14, 0.03)
Overweight/obese	0.05 (-0.03, 0.13)	0.15 (0.07, 0.24)***	-0.15 (-0.61, 0.30)	-0.13 (-0.54, 0.28)
Weight-for-Age Z-score				
All weight categories[4]	0.05 (0.01, 0.09)**	0.10 (0.03, 0.16)**	0.21 (0.11, 0.32)***	0.01 (-0.04, 0.07)
Underweight[4]	0.05 (-0.02, 0.13)	Non-estimable	0.31 (0.15, 0.47)***	0.03 (-0.06, 0.13)
Normal weight	0.07 (0.02, 0.12)**	0.14 (0.03, 0.24)**	0.30 (0.16, 0.43)***	0.02 (-0.05, 0.10)
Overweight/obese	0.05 (-0.02, 0.12)	0.12 (0.04, 0.20)**	-0.13 (-0.60, 0.34)	-0.02 (-0.32, 0.29)
**Categorical Outcomes**				
Small for gestational age				
All weight categories[4]	0.96 (0.92, 1.00)	0.83 (0.74, 0.93)**	0.87 (0.76, 1.00)	0.95 (0.90, 1.00)*
Underweight	0.93 (0.90, 0.97)***	--	--	--
Normal weight	0.93 (0.87, 1.00)*	--	--	--
Overweight/obese	0.90 (0.79, 1.03)	--	--	--
Low birth weight				
All weight categories[4]	0.94 (0.89, 1.00)*	0.80 (0.71, 0.90)***	0.76 (0.64, 0.89)***	0.99 (0.92, 1.07)
Underweight	0.91 (0.85, 0.98)**	--	--	--
Normal weight	0.92 (0.85, 0.99)*	--	--	--
Overweight/obese	0.86 (0.71, 1.04)	--	--	--

Preterm Birth				
All weight categories[4]	0.87 (0.79, 0.86)**	0.68 (0.60, 0.78)***	0.92 (0.62, 1.36)	0.98 (0.87, 1.11)
Underweight	0.89 (0.79, 0.99)*	--	--	--
Normal weight	0.88 (0.76, 1.02)	--	--	--
Overweight/obese	0.80 (0.67, 0.95)*	--	--	--

Note: Models for categorical outcomes could not be run by site and BMI due to small sample sizes.
[1]Democratic Republic of the Congo excluded due to imprecise gestational dating.
[2]Adjusted mean difference is calculated for continuous outcomes (length and weight) and adjusted relative risk for categorical outcomes (low birth weight, preterm birth, and small for gestational age.
[3]Mixed effect regression models were fit, using linear models for continuous outcomes. All models are adjusted for treatment arm, age, parity, weight gain from baseline to 12 weeks gestation, and site. * *P*<0.05, **P*<0.01, ****P*<0.001.
[4]Weight categories are defined based on BMI (kg/m^2): underweight (BMI <18.5), normal weight (BMI 18.5-24.9), and overweight/obese (BMI ≥25).

REFERENCE FOR SUPPLEMENTAL MATERIAL

INTERGROWTH-21st Project. International Standards for Newborn Size. 2018. Internet: https://intergrowth21.tghn.org/newborn-size-birth/ accessed April 2020.
